# Application of Biosensors for the Detection of Mycotoxins for the Improvement of Food Safety

**DOI:** 10.3390/toxins16060249

**Published:** 2024-05-27

**Authors:** Rafał Szelenberger, Natalia Cichoń, Wojciech Zajaczkowski, Michal Bijak

**Affiliations:** Biohazard Prevention Centre, Faculty of Biology and Environmental Protection, University of Lodz, Pomorska 141/143, 90-236 Lodz, Poland; natalia.cichon@biol.uni.lodz.pl (N.C.); wojciech.zajaczkowski@biol.uni.lodz.pl (W.Z.); michal.bijak@biol.uni.lodz.pl (M.B.)

**Keywords:** mycotoxins, biosensors, food management, detection

## Abstract

Mycotoxins, secondary metabolites synthesized by various filamentous fungi genera such as *Aspergillus*, *Penicillium*, *Fusarium*, *Claviceps*, and *Alternaria*, are potent toxic compounds. Their production is contingent upon specific environmental conditions during fungal growth. Arising as byproducts of fungal metabolic processes, mycotoxins exhibit significant toxicity, posing risks of acute or chronic health complications. Recognized as highly hazardous food contaminants, mycotoxins present a pervasive threat throughout the agricultural and food processing continuum, from plant cultivation to post-harvest stages. The imperative to adhere to principles of good agricultural and industrial practice is underscored to mitigate the risk of mycotoxin contamination in food production. In the domain of food safety, the rapid and efficient detection of mycotoxins holds paramount significance. This paper delineates conventional and commercial methodologies for mycotoxin detection in ensuring food safety, encompassing techniques like liquid chromatography, immunoassays, and test strips, with a significant emphasis on the role of electrochemiluminescence (ECL) biosensors, which are known for their high sensitivity and specificity. These are categorized into antibody-, and aptamer-based, as well as molecular imprinting methods. This paper examines the latest advancements in biosensors for mycotoxin testing, with a particular focus on their amplification strategies and operating mechanisms.

## 1. Introduction

Mycotoxins are toxic compounds produced as secondary metabolites by various filamentous fungal species. The production of mycotoxins is predominantly associated with the genera of *Aspergillus*, *Penicillium*, *Fusarium*, *Claviceps*, and *Alternaria*, and occurs under specific conditions [1,2]. Mycotoxins constitute a group of structurally diverse chemical compounds characterized by a low molecular weight, generally less than 1000 Da [3]. According to the current state of the art, there are more than 500 mycotoxins that have been defined as toxigenic and harmful to human, animal, and plant health. Each of them can be included in particular groups that have their own set of toxic effects; however, the most commonly occurring and well established include Aflatoxins (AFTs), Ochratoxins (OTs), Fumonisins (FUMs), Zearalenone (ZEN), Trichothecenes (TCTs), Patulin (PAT), and Citrinin (CT) [2]. The growth of mycotoxigenic mold can take place during any phase of cultivation, harvesting, or storage, but requires specific conditions. One of the most important requirements is temperature. For optimal growth, the temperature range spans from 20 °C to 37 °C; however, for mycotoxin production, the temperature should be within the range of 25.5 ± 5.5 °C. In addition to temperature, water activity is also a critical factor influencing mycotoxin production. Water activity denotes the quantity of water accessible for microbial and chemical processes within a substance like food [3]. It is determined by comparing the vapor pressure of water within the substance to the vapor pressure of pure water at the same temperature and pressure [4]. Its optimum varies between 0.83 aw and above. An increased humidity (70–90%) and moisture content (20–25%) are also critical factors that ensure the optimal growth and production of mycotoxins [3].

As a byproduct of the fungal metabolic processes, mycotoxins are sorely poisonous and their consumption may lead to acute or chronic health problems [5]. Intoxication with one of the above-mentioned compounds results in mycotoxicosis, whose symptoms may vary depending on the type of mycotoxin and may include the following: hepatotoxicity, nephrotoxicity, neurotoxicity, cytotoxicity, teratogenicity, and carcinogenicity (Table 1) [1].

Fungal species that produce mycotoxins may be divided into two additional groups: field fungi, which contaminate agricultural commodities before harvesting, and storage fungi, which are present after harvesting [26]. This implies that mycotoxins may manifest not solely during the cultivation phase, but that they also potentially emerge during subsequent stages such as transportation, storage, and processing. The progression of mycotoxin formation is regarded as an inherently unpredictable phenomenon, owing to the notable resilience of these compounds to elevated temperatures and various physical and chemical treatments. Furthermore, the contamination of animal feed with mycotoxins may lead to their transfer into animal-derived commodities, including milk and meat [33]. Despite the existence of numerous strategies and established protocols devised to mitigate the risk of mycotoxin occurrence and ensure their continuous surveillance throughout the food processing chain, their presence remains a substantial concern. Thus, the development of supplementary mechanisms is imperative to bolster existing preventive and diagnostic methodologies.

However, despite the application of numerous procedures and the continual refinement of existing methods, the issue of food contamination with mycotoxins persists and leads not only to the deterioration of health in many individuals worldwide, but also to significant economic losses. Therefore, the development of new methods as well as the enhancement of current processes that would help in the identification of contaminations are needed. In this review, we focus on the role of biosensors as potential tools to streamline current regulations and utilized processes and procedures. What is more, currently, in Europe, numerous strikes are occurring within the agricultural sector, which are protesting against the implementation of new EU directives that impact the restriction of cultivation in Europe. These regulations are highly stringent and may result in reduced cultivation within Europe. To meet the demand for these essential products, many countries have opted to import goods from non-European Union countries where strict regulations and principles regarding food production, storage, and transportation do not apply [34]. This poses a real threat, with an influx of mycotoxin-contaminated products not only to stores but also to animal breeders.

## 2. Current Approaches in Mycotoxins Detection

### 2.1. Sampling

According to current knowledge, the preparation of a sample for identification necessitates the implementation of procedures encompassing sampling, grinding, mixing, extraction, and purification. The contamination of natural, solid products with mycotoxins is non-homogeneous and may exhibit random distribution, potentially resulting in false-negative results and the failure to identify existing threats when sampling from inappropriate areas is conducted [35]. To ensure the best possible outcomes, Commission Regulation (EC) No. 401/2006 established standardized procedures and criteria for sampling in mycotoxin analysis, outlining methods for both, with sampling and analysis to be employed in the official control of the mycotoxin levels in foodstuffs [36,37].

### 2.2. Sample Preparation: Extraction and Purification

After sampling, the sample should be ground and mixed to allow and hasten the chemical reaction processes. Based on the available literature, the final size of the particles after homogenization should be approximately 500 µm [36,38]. There are many different approaches to extracting mycotoxin, and the proper selection of the pretreatment method is crucial due to the various consistencies of food products. Based on the traditional techniques, the most commonly selected are Solid–Liquid Extraction (SLE), Solid-Phase Extraction (SPE), and QuEChERS.

SLE is one of the most common methods used to extract mycotoxins from different foodstuffs. SLE is easy to perform and does not require large financial outlays and any specific instruments. However, to obtain accurate and specific results, the solvent must be carefully selected [39]. Huertas-Pérez et al. showed that the same solvent for two different rice types (white and brown) does not work with the same recovery ratio; thus, 100% acetonitrile (ACN) may be used for AF detection in SLE in white rice, but not in brown rice [40].

Solid-Phase Extraction (SPE) is an effective method used to extract mycotoxins. The liquid sample that contains the analytes of interest is passed through the unique cartridge or disk that contains high-affinity adsorbent particles. Further, the analyte interacts with the sorbent, enabling other interfering compounds to be washed. In the last stage, the analyte of interest is eluted from the sorbent by a selected solvent or mixture of solvents [41]. In contrast to other conventional techniques, SPE boasts reduced solvent usage, an efficient concentration, and enhanced recovery rates [39]. In addition to these advantages, SPE also has some disadvantages. The crucial limitation is that for detection of different mycotoxins, various sorbents are necessary; thus, there is no one cartridge for all mycotoxins. Furthermore, the analyte recovery efficiency may vary based on the solvent type, ionic strength, and pH of the sample [42].

For extraction and purification, one of the currently used procedures is QuEChERS, whose name comes from the following terms: Quick, Easy, Cheap, Effective, Rugged, and Safe [43]. This methodology encompasses two main techniques: liquid–liquid distribution, in which ACN is mainly used for extraction, and dispersive solid-phase extraction (d-SPE), with a specific sorbent used for the purification of the obtained extract. Like in the previously described methods, the key to the good optimization of QuEChERS is a good selection of sorbents [39].

Leite et al. performed a study in which a comprehensive assessment of SLE, SPE, and QuEChERS was performed in maize. Based on the obtained results, the authors suggested that the methodology based on QuEChERS, with C18 as the sorbent, had the best performances when they performed a comparison [44].

### 2.3. Methods Used for Mycotoxin Detection

Since the initial discovery of mycotoxins, numerous analytical methods have been explored and utilized for assessing their presence in food and feed. Chromatographic techniques have predominantly been employed, owing to their versatility; these include thin-layer chromatography (TLC) and high-performance liquid chromatography (HPLC), in conjunction with a range of detectors such as diode array, UV, and fluorescence, gas chromatography–tandem mass spectrometry (GC-MS/MS) and liquid chromatography–tandem mass spectrometry (LC-MS/MS) [45]. However, it is also worth noting the significant role of antibody-based immunoassays in mycotoxin identification.

TLC is a type of liquid chromatography that was widely used between the 1980s and 2000s. Despite the existence of much more specific and sensitive detection methods, its major advantage is its low analysis cost, meaning that TLC is still in use today. TLC utilizes a stationary phase, typically composed of silica, alumina, or cellulose, affixed to an inert substrate such as plastic or glass that holds the analyte in place during separation. Meanwhile, the mobile phase, usually comprising methanol, acetonitrile, and water mixtures, transports the sample across the solid stationary phase [46]. The visualization of TLC can be divided into three main categories: destructive, semi-destructive, and non-destructive. This division depends on whether the separated molecule is transformed into another chemical compound that allows visualization or if the compound remains unchanged during the staining process. Destructive methods include chemical staining with a range of different chemical compounds such as ninhydrin, bromocresol green, and p-methoxybenzaldehyde. Iodine staining is classified as a semi-destructive method. Due to the fact that the color change is not always permanent, the stained spots should be marked with a pencil, for example, to avoid losing the results. Non-destructive methods include UV staining at wavelengths of 254 nm (short wavelength) or 366 nm (long wavelength). In this type of staining, chromophores block UV light absorption, creating black spots on a bright background [47].

In the study conducted by Overy et al., TLC was used to assess the mold growth of *C. sativa* in grocery stores in Canada [48]. Furthermore, in the study by Aiko et al., the TLC method showed that out of 63 samples of medicine herbs, 58 were contaminated by mycotoxins [49].

To overcome the limitations of TLC, more specific, sensitive, and automated techniques were developed, including liquid chromatography (LC). LC allows for the simultaneous identification of numerous mycotoxins, irrespective of their chemical composition and biological activity. LC is characterized by a significantly greater specificity and effectiveness in identifying mycotoxins compared to TLC; however, it requires significantly greater financial investment, including the purchase of appropriate equipment [50]. High-performance liquid chromatography (HPLC) is a gold-standard method in the analysis of mycotoxin contamination in various food products. The protocols for their detection largely follow similar methodologies, using UV–visible or fluorescence detectors or even mass spectrometry to improve the effectiveness and sensitivity [45]. HPLC was used to detect AFB1, CIT, and OTA in rice samples [51], OTA in wine [52], and patulin in apple juice [53], among many other foodstuffs, emphasizing the efficiency of HPLC in mycotoxin detection [45].

Chromatography (GC) is another type of chromatography in which the mobile phase is gas. GC is not regularly used for the identification and quantification of mycotoxins in food samples due to the need for the derivatization of mycotoxins, which are not mostly volatile. Thus, it is not usually expected that a commercial protocol for GC will be obtained, especially with the presence of faster and cheaper methods like HPLC [45]. However, some studies have used GC for the identification of mycotoxin in food products. In the study conducted by Rodríguez-Carrasco et al., T-2 was found in wheat-based cereals, patulin was found in rice-based cereals and ZEA was identified in maize-based cereals [54]. Furthermore, deoxynivalenol and diacetoxyscirpenol were found in wheat semolina.

One of the most commonly used antibody-based immunoassays for mycotoxin detection is the enzyme-linked immunosorbent assay (ELISA). ELISA enables the fast, simple, reliable, and simultaneous analysis of many samples. ELISA kits are usually based on a competitive assay format in which a primary antibody binds to a specific molecule, or is enzyme-conjugated. Upon formation of the complex, interaction with a chromogenic substrate yields a measurable outcome. Besides its portability, fast execution time, and high specificity, ELISA kits are designed for single use, which may increase the cost of performing a screening analysis of many mycotoxins [42,45]. Furthermore, whether monoclonal or polyclonal antibodies are used, the detection sensitivity may vary. In the study conducted by Omar et al., ELISA was used to detect AFB1, AFB2, AFG1, and AFG2 in wheat samples [55], OTA and ZEA in different types of tea [56], and AFB1, OTA, and ZEA in corn samples [57].

Another type of antibody-based immunoassay that is used to detect mycotoxins in various samples is the lateral flow immunoassay (LFA). LFA is a low-cost, simple, paper-based test for the rapid identification and quantification of various analytes in mixtures of substances. The procedure involves the use of capillary beds resembling porous paper fragments to transport the analyte, while unique elements responsible for the recognition of the analyte of interest attach to immobilized moieties on the membrane surface [42,58]. LFA has been used in many studies focused on the detection of mycotoxins in different food products. In the study conducted by Jin et al., diacetoxyscirpenol was found in rice [59]. What is more, in Li et al.’s study, ZEA, T-2, and DON were detected in maize [60].

## 3. Mycotoxin Detection Techniques Based on Biosensors

### 3.1. Sensing Strategy for Mycotoxin Analysis

A biosensor is an advanced analytical tool consisting of bioreceptors, such as enzymes, antibodies, organelles, tissues, and cells, coupled with various transducers, including electrochemical, optical, piezoelectric, and thermal components, depending on the specific design of the biosensor. The bioreceptor segment, also known as the detector element, possesses a selective site capable of recognizing the target analyte and potentially converting it into products. Transducers play a critical role in converting the bioreceptor-mediated biological recognition event into an observable and quantifiable electrical, optical, mechanical, or thermal signal. The magnitude of this signal is directly proportional to the concentration of a particular analyte or set of analytes of interest [61].

Biosensors can be categorized into various classes depending on the type of transducer utilized. The type of material used, the specification of the sensing device and the actual signal conversion mechanism determine the transducer needed for a biosensor. The design of the device also plays an important role in defining the final specifications, although the transducer specifications are mainly defined by the capabilities of the active sensing material. To this end, the transducer mechanism simply defines the biosensor class, e.g., a biosensor is classified as an ‘electrochemical biosensor’ if it uses an electrochemical transducer [61].

They can be further divided into different categories based on their transduction mechanisms. The conversion of a biochemical signal into an electrical signal is achieved through various techniques including voltammetry (which measures voltage), amperometry (which measures current), potentiometry (which measures potential or charge accumulation), impedimetry (which measures impedance encompassing both resistance and reactance), and conductometry (which measures changes in the conductive properties of a medium) [62].

Generally, electrochemical biosensors are structured as either three- or two-electrode systems. A three-electrode system comprises one working electrode, one counter electrode, and one reference electrode. In the case of a two-electrode configuration, the reference and counter are short-circuited to form the so-called electrode reference (counter) while the working electrode remains unchanged [63].

Among the several optical detection methods, such as surface plasmon resonance (SPR) and optical waveguide light spectroscopy (OWLS), fluorescence sensing techniques have attracted huge interest due to their easy operation, short analytical time, and convenient signal reading [64].

Fluorescence, a phenomenon involving the absorption and subsequent re-emission of photons by atoms or molecules at longer wavelengths, forms the basis of fluorescence sensors. These sensors detect target analytes either through their inherent fluorescence or via conjugation with a fluorophore, a molecule capable of emitting light upon excitation [65].

Fluorescence-based biosensors operate primarily by amplifying (“turn-on”) or quenching (“turn-off”) fluorescence signals mediated by the presence of the target analyte, sometimes in conjunction with a fluorophore. Optical methods using fluorescence properties offer the precise identification and quantitative measurement of food contaminants in a variety of media, including food products, plants and water [65].

Fluorescent sensors are characterized by their affordability, sensitivity, and fast response time [66]. They are therefore used in the construction of toxin detection devices in food, biomedical research, medical diagnostics, pathogenic bacteria testing, and environmental monitoring [67,68,69].

Electrochemiluminescence, also called electrogenerated chemiluminescence (ECL), is another type of sensor that demonstrates its potential advantages in comparison to other optical techniques. ECL is a type of luminescence produced during electrochemical reactions in solutions. The main advantage of this method is that there is no need for an excitation light source. This streamlines the measurement system and negates the impact of contamination, thereby reducing the influence of background luminescence impurities and scattered light. ECL reactions are potential-controlled, rendering them straightforward operations. Therefore, ECL technology represents an optimal analytical instrument for the identification of trace toxins in foodstuffs, the environment, and the diagnosis of disease, etc. Depending on the types of sensing elements employed, ECL biosensors can be categorized into three groups, namely those based on antibodies [70,71,72], aptamers [73,74,75], as well as molecular imprinting polymers (MIPs) [76,77,78]. The development of these types of detectors is the focus of the rest of this paper.

**Figure 1:**
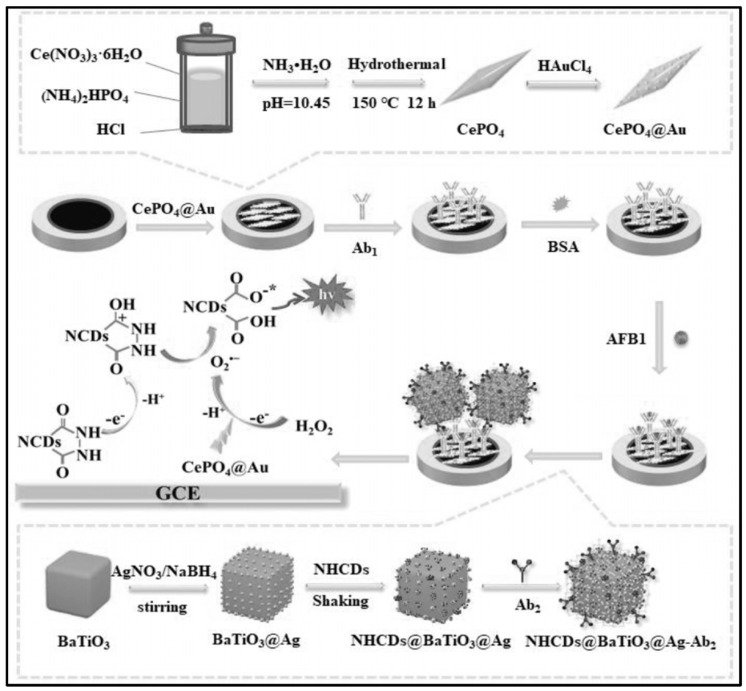
Sandwich-type ECL immunosensor for AFB1 detection (Reproduced with permission from [79]).

Immunosensors, utilizing antibodies as recognition elements, demonstrate heightened sensitivity and specificity in contrast to conventional detection techniques like nucleic-acid-based methods. This arises from the unique characteristics of the binding between antigens and antibodies. Furthermore, the functionality of these sensors is comparatively straightforward, enabling their miniaturization, automation, and commercialization with ease. Research conducted by Li’s team demonstrated a highly sensitive multi-layered ECL immunosensor for the detection of Aflatoxin B1 (AFB1) in corn, rice, and wheat. First, they use in the immunosensor a new type of co-reaction accelerator based on Ce^3+^/Ce^4+^ redox pairs, namely cerium phosphate@gold (CePO_4_@Au) (See Figure 1). CePO_4_@Au was applied as a modification on a glassy carbon electrode (GCE), facilitating the creation of a spacious and uniform surface for the attachment of the target antibody for detection. Moreover, the ECL signal is enhanced due to the capability of the employed technology to decompose H_2_O_2_ into superoxide radicals, which then react with nitrogen-doped hydrazide-conjugated carbon dot radicals (NHCDs^•^). For additional signal amplification, they synthesized BaTiO_3_@Ag nanoparticles with high conductivity. These nanoparticles provided anchoring sites for immobilizing another antibody (Ab2) and served as efficient carriers for modifying NHCDs. The biosensor provided the accurate detection of AFB1 with a sensitivity range of 0.01 pg/mL–100 ng/mL [79].

Li et al., using a coated Au electrode made of CaCO_3_ that has excellent electrochemical properties and acts as a scaffold for Ru(bpy)_3_^2+^, were able to detect Ochratoxin A in coffee beans. They utilized the porous hierarchical structure of eggshells as a template for the preparation of Au/CaCO_3_. This dual purpose allowed the capturing of Au ions, preventing nanoparticle aggregation while also offering a substantial surface area for immobilizing the ECL reagent (Ru(bpy)_3_^2+^). The subsequent steps involved OTA biosensor preparation by modifying Au/CaCO_3_, (Ru(bpy)_3_^2+^), and the antibody on GCE. When the target mycotoxin is present, it becomes entrapped by the sensing interface, leading to a notable decrease in the ECL intensity. This diminishment is a consequence of OTA’s inhibition of interfacial electron transfer. This biosensor demonstrates the capability to measure OTA within a linear range spanning from 10 pg/mL to 100 ng/mL, boasting a relatively low detection limit of 5.7 pg/mL [80,81].

However, there are still limitations and challenges to be addressed in the use of immunosensors. Antibody bioactivity is susceptible to environmental conditions, and the potential for cross-reactions between antibodies and other biomolecules is notable. This phenomenon leads to inaccurate results, including both false positives and false negatives during real sample analysis. Therefore, it is imperative that the accuracy and reliability of detection are further improved in future studies and developments.

As the technologies and methods advance, improvements in the selectivity, sensitivity, and stability of immunosensors are expected to be achieved. This development will significantly contribute to reliable and accurate mycotoxin detection, benefiting various fields including food safety and healthcare.

### 3.2. Aptamer-Based Biosensor

Aptamers are short, single-stranded DNA or RNA (ssDNA or ssRNA) molecules that can selectively bind to a specific target, including proteins, small molecules, toxins, and even live cells. In contrast to antibodies, aptamers are not sensitive to temperature and are chemically stable; therefore, they have a simple storage condition allowing for long-term storage. To date, a number of DNA hybridization techniques have been employed in aptamer-based biosensors with the objective of enhancing their analytical performance. These include rolling circle amplification (RCA) [81], loop-mediated isothermal amplification, hybridization chain reaction (HCR) [82,83], and DNA walkers [84,85], among others. The likelihood of coupling nanoparticles offering unique physical and chemical properties to the terminal of DNA for enhanced biosensor ECL performance is promising. This potential has drawn considerable attention towards aptamer-based biosensors for mycotoxin quantitative detection.

**Figure 2:**
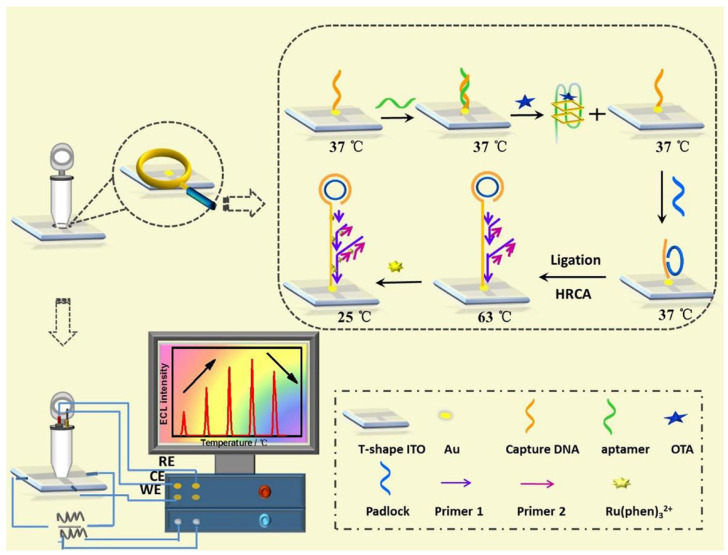
ECL biosensor using hyperbranched RCA strategy for the in situ monitoring of OTA (Reproduced with permission from [84]).

A research team led by Lin et al. demonstrated a sensitive ECL biosensor for the real-time monitoring of Ochratoxin A (OTA) in red wine, leveraging a hyperbranched RCA strategy (Figure 2) [84]. DNA was immobilized onto an Indium Tin Oxide (ITO) electrode, followed by aptamer hybridization under a 37 °C electrode temperature. The presence of OTA toxin led to the formation of an OTA–aptamer complex. This complex then activated DCA, triggering the formation of long DNA sequences, as well as double-stranded DNA. This enabled the intercalation of numerous ECL molecules (Ru(phen)_3_^2+^) into the dsDNA’s groove, leading to appreciably amplified ECL signals. Ultimately, the biosensor served as a sensitive OTA detector with a wide linear range of 0.075–10 pg/mL and a lower limit of detection (LOD) of 8 fg/mL at 65 °C.

In a separate study, Yuan et al. used the loop-mediated isothermal amplification technique (LAMP) to build a biosensor for the detection of the OTA toxin [86]. Prior to OTA exposure, aptamer-capturing DNA reactions resulted in dsDNA. Subsequently, the introduction of the OTA toxin activated the remaining aptamers, triggering the LAMP technique, allowing Ru(phen)_3_^2+^ to intercalate into dsDNA. This resulted in large cumulative ECL molecules on the sensing surface, significantly amplifying the electrogenerated chemiluminescence signal. Ultimately, the biosensor served as a high-sensitivity OTA detector with a wide linear range of 0.00005 nM–100 nM and a lower limit of detection (LOD) of 10 fM.

**Figure 3:**
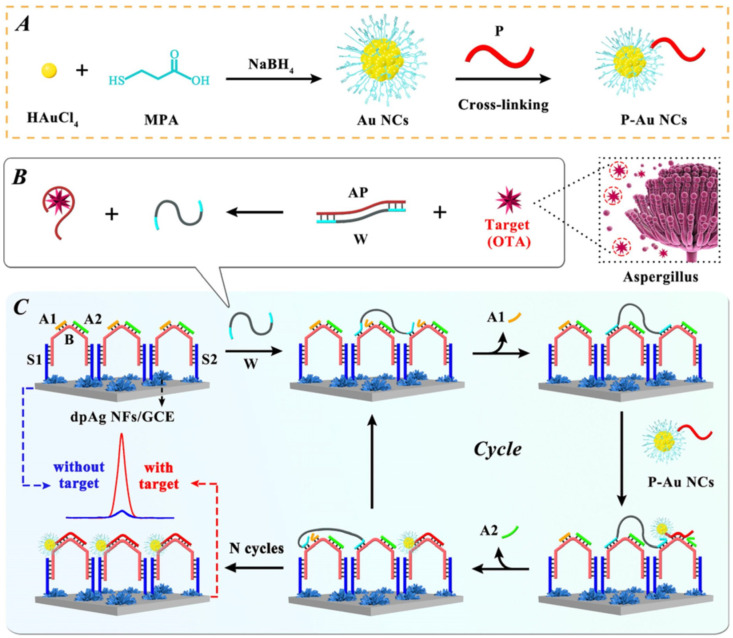
Non-enzymatic ECL biosensor for the detection of OTA based on the efficient amplification strategy of DNA walker. (**A**) Preparation of monodispersed MPA-Au NCs. (**B**) The target transformation process. (**C**) Schematic diagram of the proposed ECL biosensor for OTA detection. (Reproduced with permission from [85]).

Shi et al. looked closer at the detection of OTA toxins in corn oils and developed a novel and simple, ultrasensitive, non-enzymatic electrochemiluminescence (ECL) biosensor, implementing an effective and efficient DNA walker amplification strategy (Figure 3) [85]. Gold nanoclusters (gated nano-capsules) were used as ECL signal probes and labelled at the DNA P terminus. The release of the bipedal DNA walkers enabled them to move independently along the DNA distributed on the electrode surface, thus facilitating the highly sensitive detection of OTA. The proposed non-enzymatic ECL biosensor can detect selectively for OTA over other mycotoxins in a linear range of 10 fg/mL up to 100 ng/mL, with a detection limit of 3.19 fg/mL.

Wei et al. developed and demonstrated a simple and sensitive method for the detection of OTA toxins in beverages such as wine and beer. The presence of Ochratoxin A is determined by the electrochemiluminescence resonance energy transfer (ECL-RET) between Cy5 and CdS QDs, and a nicking endonuclease-driven DNA walker [87]. The presence of OTA in target samples increases the ECL energy transfer from the CdS QDs to the Cy5, significantly increasing sensitivity and allowing the detection of OTA in the range of 0.05 nM to 5 nM, with a LOD of 0.012 nM.

On the other hand, Jia et al. demonstrated a novel approach using a label-free, small-molecule and nanoparticle strategy to detect OTA in herbs, based on lily and rhubarb [88]. The proposed strategy is based on the coating of a gold electrode with chitosan/CdSe@CdS quantum dots, which exhibit good biocompatibility and strong ECL intensity. The glutaraldehyde additives help to cross-link the OTA aptamers formed on the electrode surface, which significantly inhibits the ECL intensity [88]. Despite the high sensitivity, specificity, and low batch-to-batch variance of aptamer-based biosensors, they also have some inherent drawbacks, including a laborious and time-consuming electrode assembly process that necessitates the optimization of the assembly conditions, such as the degradation of the biological-based materials at the sensing electrode interface over time, resulting in the insufficient long-term stability of the modified electrode.

A regenerative biosensor for aflatoxin B1 (AFB1) based on the quenching effect of ferrocene (Fc) on the ECL of a graphitic carbon nitride (g-CN) detector was developed in an innovative approach by Tian et al. [73]. The ferrocene-labeled aptamers were altered on a g-CN/GCE surface. The addition of the AFB1 target induced the aptamer to change its conformation, significantly enhancing the quenching efficiency of Fc on the ECL signal of the g-CN. In addition, oxidized AFB1 could also react with the high-energy state of g-CN, further reducing the electrochemiluminescence signal. Thanks to its quenching effect at the sensing electrode, the biosensor successfully detected AFB1 in the range of 0.005 ng/mL to 10 ng/mL. One of the most remarkable features of this biosensor was its ability to regenerate when the temperature was increased to 40 °C, with less than 5% variation in the results within five regeneration cycles.

Recently, the development of ratiometric biosensors has also aimed to reduece false-positive responses [89,90]. The ratio between two different signals can offer a self-calibration function, improving the accuracy of the biosensor. Sensitivity could be significantly improved if these two signals showed an inverse change in response to the target. Looking forward, novel biosensors are expected to overcome the inherent limitations of existing aptamer-based biosensors, such as the exhaustive electrode assembly process and the gradual degradation of the sensing interface over time, in order to improve the long-term stability. Therefore, continuous efforts are being made to optimize these sensing devices for better practical applications.

**Figure 4:**
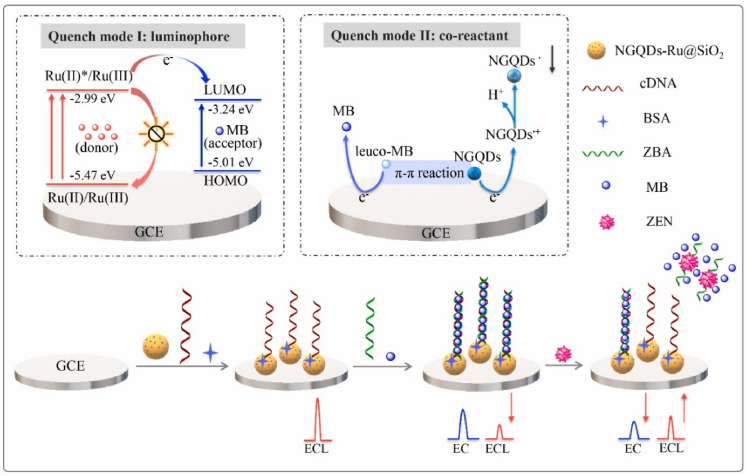
ECL–electrochemical ratiometric aptasensor based on the dual quenching effects of MB (Reproduced with permission from [91]).

This enabled the determination of zearalenone (ZEN) (Figure 4), based on the dual quenching effects of methylene blue (MB) [91]. This feat was achieved by using Ru@SiO_2_ or Ru(bpy)_3_^2+^-doped silica nanoparticles as the ECL donor. As a self-enhancing co-reactant, nitrogen-doped graphene quantum dots (NGQDs) were then modified on the Ru@SiO2 surface. In the presence of ZEN, the dsDNA on the NGQDs-Ru@SiO_2_/GCE surface underwent deformation, resulting in the release of MB from the electrode surface and the recovery of the ECL signal. An extremely low detection limit of 0.85 fg/mL was achieved by exploiting the ratio between the “turn-on” ECL signal and the “turn-off” electrochemical signal of MB.

Jie et al., by controlling the self-assembly properties of a DNA nanotube (DNANT) in the presence of Ru(phen)_3_^2+^ and methylene blue (MB), were able to present a dual ECL and electrochemical biosensor for the detection of Dam methylase (MTase) and aflatoxin B1 (AFB1) in food samples. In this strategy, the MB and Ru(phen)_3_^2+^ molecules embedded in the dsDNA on the DNA nanotube were used to generate a detection signal and an electrochemical signal, respectively [92]. Despite their high sensitivity, selectivity and low batch-to-batch variability, the manufacture of aptamer-based biosensors presents a number of challenges. In particular, the process of assembling the electrodes can be laborious and time consuming. Importantly, the binding at the sensing interface weakens over time, resulting in modified electrodes that lack long-term stability. Yet, advancements continue to be made in the exciting and promising field of aptamer-based biosensors, with the ultimate goal of improving existing systems, making them more user-friendly, robust, reliable, and suitable for long-term applications. With each discovery, we move a step closer to making this a reality.

### 3.3. Molecular Imprinting

In addition to the aforementioned antibody and aptamer recognition techniques, molecular imprinting-based methods have gained substantial attention for their distinctive advantages, including simplicity, rapidity, reusability, and exceptional selectivity. Molecularly imprinted polymers (MIPs) play a crucial role in these recognition processes by creating specific cavities that mimic the shape and structure of target molecules, thereby offering high selectivity towards analytes with similar attributes to the template molecules. These unique characteristics have led to the utilization of MIP strategies for the label-free detection of mycotoxins, showcasing their efficacy in biosensing applications [93,94].

Zhang et al. suggested the use of a molecularly imprinted electrochemiluminescence (MIP-ECL) sensor device utilizing Ru@SiO_2_ NPs, which is used as an ECL emitter for signal amplification. By encapsulating a high concentration of ECL molecules (Ru(bpy)_3_^2+^) within SiO_2_ NPs, the developed MIP-based ECL biosensor demonstrated exceptional sensitivity and selectivity for detecting FB1, achieving a notably low detection limit of 0.35 pg/mL. Furthermore, Zhang et al. and collaborators used ECL resonant energy transfer to detect OTA, with MIP as a sensing element. The Ru(bpy)_3_^2+^-doped silica NPs were utilized as ECL donors, while CdTe QDs acted as ECL acceptors to amplify the ECL intensity for OTA detection. The specific cavities within MIPs not only facilitated target recognition but also served as conduits for co-reactant transfer to the electrode surface, activating the ECL signal. The biosensor exhibited dual-quenching effects upon target binding, resulting in a wide linear detection range of 1.0 × 10^−5^ to 11.13 ng/mL and an impressively low detection limit of 3.0 fg/mL [95].

Li et al. introduced a silica-encapsulated MAPB QDs@SiO_2_-based MIP-ECL sensor for AFB1 detection in corn oil samples, showcasing improved ECL signal amplification through the encapsulation of the luminophore in SiO_2_ particles. This innovative biosensor achieved the sensitive and label-free detection of AFB1, with an ultra-low detection limit of 8.5 fg/mL. The comprehensive evaluation of the biosensor using corn oil samples displayed high recoveries and excellent long-term storage stability, affirming its robust performance in real sample analysis [96].

Furthermore, the integration of metal–organic frameworks (MOFs) in the construction of MIPs has garnered significant interest due to their adjustable three-dimensional structures and ease of functionalization. Wei and collaborators embedded CH_3_NH_3_PbBr_3_ QDs in ZIF-8 MOFs to develop a highly stable imprinted polymer for FAB1 detection. Encapsulating the QDs in ZIF-8 MOFs significantly improved the stability of the ECL by reducing external disturbances and preventing the QDs from dissolving. The imprinted polymer membrane based on QDs@ZIF-8/GCE resulted in enhanced sensitivity, resulting in a wide linear detection range of 11.55 fg/mL to 20 ng/mL for FB1 and a low detection limit of 3.5 fg/mL. Comparative analysis with HPLC demonstrated the superior sensitivity of the MIP-based biosensor [87].

MIPs offer clear advantages, including benign storage conditions, long-term usability and cost-effectiveness. However, they do have some drawbacks, including an average sensitivity, a limited ability to recognize macromolecular targets, and complex preparation processes. Despite these challenges, continued advances in MIP technology will undoubtedly enhance the capabilities of biosensors and meet analytical needs across industries. It is clear that continued research efforts will drive the development of highly efficient MIP-based biosensors for a variety of applications. This is part of the ongoing evolution and innovation in biosensor technology. MIP-based biosensors are a pivotal advancement in analytical chemistry, specifically in mycotoxin detection. They offer high selectivity, sensitivity, and an impressively low detection limit due to their unique recognition abilities. These biosensors are beneficial in various sectors including food safety, environmental monitoring, and clinical diagnostics.

The future improvement and optimization of MIP-based biosensors are expected through the incorporation of innovative materials and advanced detection techniques. The goal is to break through existing limitations and enhance their role in handling complex detection issues, particularly concerning mycotoxins.

The merging of molecular imprinting and advanced biosensing techniques promises to transform analytical chemistry, delivering precise and trustworthy detections in various sample types. Sustained interdisciplinary collaboration among researchers is crucial for further the development and wider adoption of MIP-based biosensors.

## 4. Integrated Biosensor Applications in Food Safety Management

As per the definition provided by the Food and Agriculture Organization of the United Nations (FAO), food safety pertains to the assurance that food, when prepared and consumed as intended, does not inflict harm upon the consumer [97]. Legislation concerning food safety in developed nations is extensively harmonized, constituting a cornerstone of health protection. This system comprises a set of regulations designed to uphold the highest standards, safeguarding the health and economic interests of consumers [98]. Additionally, it includes established control standards addressing the hygiene of food and food products, animal health and welfare, plant health, and the prevention of the risk of contamination with external substances [97]. In the realm of ensuring the safety of food, producers bear the responsibility of mitigating risks to consumers through their actions. The overarching principle is to employ an integrated approach, encompassing all facets of the food chain, from farm to fork. Adherence to food safety necessitates the thorough and systematic management of food hygiene and standards, ensuring that the food products offered are deemed safe for consumption [99]. In the European Union, the implementation of an integrated food safety policy involves various activities. These activities encompass ensuring the effectiveness of control systems and evaluating compliance with standards in food safety and quality, animal health, animal welfare, and animal nutrition and health. Collaborating with the European Food Safety Authority (EFSA) contributes to establishing a scientific basis for risk management [97,100].

Food products originating from natural sources inherently harbor microbiological risks. Despite significant advancements in this field, microbiological factors persist as the primary threat to food safety. Microbiological criteria serve as guidelines regarding the acceptability of food products and the procedures involved in their production [101]. Preemptive measures, such as implementing Good Hygiene and Manufacturing Practices (GHP, GMP), coupled with applying the principles of Hazard Analysis Critical Control Points (HACCP), are pivotal in ensuring food safety. It is imperative to acknowledge that exclusive reliance on microbiological testing cannot unequivocally guarantee the safety of the tested food items. However, these criteria establish specific targets and benchmarks, providing invaluable guidance to both food businesses and authorities in their respective roles of managing and monitoring the safety of food products [102].

Community food legislation strives to achieve a reasonable equilibrium between the risks and benefits associated with intentionally used substances and pollution mitigation [103]. Regulations concerning pollutants are founded on scientific guidance and the principle that pollutant levels should be minimized to the greatest extent feasible through sound work practices. Maximum levels have been established for specific contaminants, including mycotoxins, to safeguard public health [97].

The incorporation of Critical Control Points (CCPs) throughout the food supply chain is a fundamental aspect of food safety management. Identifying CCPs allows for targeted control measures to be implemented at specific stages of production, processing, and distribution, where the risk of mycotoxin contamination is significant. Legislative changes and an increased awareness of mycotoxin threats play a pivotal role in driving these efforts [104,105,106]. Implementing control measures should start at the manufacturer’s level and extend throughout the entire supply chain, including storage facilities, transportation, processing, and distribution. This comprehensive approach ensures that mycotoxin contamination risks are addressed at every stage, ultimately safeguarding the quality and safety of the final food products reaching consumers [104,107]. Furthermore, developing and implementing plans and procedures for mycotoxin protection should be a priority for all participants in the food chain. This includes the companies and employees involved in cleaning services throughout the food processing cycle. Regular monitoring, testing, and adherence to established protocols are essential components of an effective food biosecurity strategy. These pivotal procedures encompass risk assessment, risk management, risk reporting, and risk communication [107,108,109].

The process of risk analysis follows a structured sequence beginning with risk assessment, encompassing the evaluation of susceptibility to food contamination. This assessment involves the formulation of a detailed action plan under the umbrella of preparedness, followed by the establishment of a food defense/protection management plan within the framework of risk management [108,110]. This procedure necessitates the integration of experimental toxicological data, along with the insights derived from several years of monitoring and controlling mycotoxin contamination in food products through validated quantitative methods. It is noteworthy that a single food product or portion may harbor more than one type of mycotoxin. A significant challenge lies in the fact that much of the available empirical toxicological observations predominantly focus on the individual health effects of specific toxins. A notable research gap exists concerning the cumulative effects resulting from the concurrent presence of multiple mycotoxins [111,112,113]. In the assessment of risk, it is imperative to recognize that the distribution of mycotoxins within a product is not uniform, posing challenges in the collection of representative samples for analysis. The French Agency for Food Safety (AFSSA) has proposed an innovative approach to enhance sample representativeness by incorporating the final product into the sampling procedure [36]. While developing a biosensor capable of identifying the sample collection site appears to be a highly challenging endeavor given the current state of knowledge, it holds significant potential and merits serious consideration.

HACCP is a systematic scientific approach designed for the analysis of recipes or any food preparation process to pinpoint CCPs. Originally developed by NASA to monitor and ensure the safety of food consumed by astronauts, the HACCP system’s efficacy led to its incorporation into the Food Code [114,115]. Comprising seven distinct steps, the HACCP system begins with the analysis of potential threats, encompassing considerations related to mycotoxin contamination. The second step involves the identification of CCPs within the process that can be controlled to prevent contamination. Subsequently, an optimized protocol is established within this system to specifically address and prevent contamination at these CCPs [102,105,114,115]. In addition to the above steps, the HACCP system mandates ongoing scrutiny of its own effectiveness and the strict maintenance of well-defined documentation. This comprehensive approach ensures the continual evaluation and refinement of the system, contributing to the overall quality and safety of the food production process.

As previously mentioned, the agri-food industry in the EU and other highly developed countries is subject to strict regulations to ensure the safety and quality of food products. The implementation of innovative solutions, such as biosensors, in the field of microbiological diagnostics plays a crucial role in meeting these regulatory standards.

Biosensors offer several advantages that make them valuable in enhancing the HACCP system. Some key advantages of biosensors in this context include simplicity of operation, low operating cost, rapid detection, on-site testing, continuous monitoring, as well as integration with HACCP systems. Biosensors are designed to be user-friendly and require minimal training for operation. This simplicity makes them accessible to a wide range of users, from food producers to inspectors. Biosensors often have lower operational costs compared to traditional laboratory methods. This cost-effectiveness is particularly beneficial for small and medium-sized enterprises in the agri-food industry, helping them comply with regulations without significant financial burden [116]. Biosensors can provide rapid results, allowing for the real-time monitoring of microbial contamination in food products. This quick turnaround is crucial for preventing the distribution of contaminated products and minimizing potential health risks [117]. The portability of some biosensors enables on-site testing, eliminating the need to transport samples to off-site laboratories. This feature is advantageous for quick decision-making and reducing the time it takes to address potential issues in the production process [118]. Biosensors can be designed for continuous monitoring, providing a more comprehensive understanding of the microbial status throughout the entire production chain. This continuous feedback allows for proactive measures to be taken to maintain food safety. Biosensors can be seamlessly integrated into the HACCP system, enhancing its effectiveness by providing real-time data and enabling more precise control over critical points in the production process [119]. By incorporating biosensors into microbiological diagnostics, the agri-food industry can strengthen its ability to detect and prevent microbial contamination, ensuring that food products meet the stringent safety and quality standards set by regulatory bodies in the EU and other developed countries. This, in turn, contributes to the overall improvement of food safety and consumer confidence in the industry [120].

The vulnerability of cereals, feed, grains, and other looses products to mycotoxin development in conditions of heat and humidity underscores the importance of monitoring at critical points in the supply chain. Implementing biosensors in screw feeders for continuous monitoring is a practical and effective solution. This approach enables the identification of mycotoxin contamination at an early stage, providing an opportunity to take immediate corrective actions before the goods are distributed further into the supply chain. Mounting prototype devices at outlets allows for the capture of batches containing mycotoxins, preventing their spread and ensuring the overall quality of the supply chain. The versatility of this application extends to the various branches of the agri-food industry involved in goods transshipment, including transport companies, purchase centers, processing plants (such as mills and the oil industry), and coffee roasteries. This not only addresses regulatory compliance, but also enhances the reputation of businesses in the industry by ensuring the delivery of safe and high-quality products.

Moreover, the idea of incorporating biosensors into collective packaging for fruits and vegetables is innovative. Placing biosensors at the bottom of the packaging to detect mycotoxins in fresh produce and nuts adds an additional layer of safety and quality assurance. This approach could be particularly valuable for consumers, retailers, and producers alike, as it provides real-time information about the freshness and safety of the products. Overall, these proposed applications of biosensors in the agri-food industry demonstrate a forward-thinking approach to addressing the challenges related to mycotoxin contamination. Continuous monitoring at key points in the supply chain, coupled with rapid detection capabilities, has the potential to significantly improve food safety standards and ensure the delivery of high-quality products to consumers.

Mycotoxins can enter the food supply chain through various routes, including plant-based products, cereals, fruits, and animal feeds. Animal products such as milk, eggs, meat, or offal can also be contaminated through the animals’ diet. This highlights the need for a comprehensive approach to monitoring and controlling the presence of mycotoxins [121]. The use of biosensors in meat processing plants, including slaughterhouses and dairies, can play a crucial role in early detection and prevention. By monitoring the presence of mycotoxins, these biosensors can help ensure that meat and dairy products meet stringent safety standards before reaching consumers. In summary, biosensors represent a significant advancement in securing the food supply chain and mitigating the risks associated with mycotoxin contamination. Their application in meat processing plants is a proactive step towards ensuring the safety of both human and animal consumption, contributing to a healthier and safer food supply for society at large.

## 5. Conclusions

The continuous advancement and integration of biosensors into food safety systems signifies a dynamic and evolving field, holding immense potential to enhance the safety and quality of the food supply chain. Progress in technology, connectivity, and interdisciplinary collaboration will further bolster the expansion of biosensor applications in safeguarding food safety.

## Figures and Tables

**Table 1:** Summary of the toxicity of the most important mycotoxins.

Mycotoxin	Toxic Effect	Species	Ref
**Aflatoxin B1 (AFB1) and B2 (AFB2)**	Strongly hepatotoxic, immunosuppressive, and carcinogenic. Mostly linked with hepatocellular carcinoma (HCC). Its metabolite (AFBO—AFB1-exo-8,9-epoxide) possesses a high affinity for forming DNA adducts, stimulates mutations, induces oxidative stress, and leads to a loss of cell integrity. AFBO inhibits basic metabolism, nucleic acid synthesis, protein synthesis, and DNA repair, thus leading to a disruption of vital cell functions.	***Aspergillus*** (*mainly A. flavus*, *A. parasiticus*, *A. nomius*)	[3,6]
**Aflatoxin G1 (AFG1) and G2 (AFG2)**	Induce atrophy of spermatogenic tubules and upregulate the expression of cyclin D1 at the mRNA and protein level in spermatocytogenic and spermatogenic cells in albino mice (20 µg/kg of AFG1). Stimulate gastric inflammation and DNA damage in mice after oral administration (100 µg/kg of AFG1). In the human GES-1 cell line, AFG1 leads to oxidative stress and DNA damage via Cytochrome P450 (particularly CYP2E1) through NF-ĸB). In Wistar Rats, the administration of AFG1 (2 mg/kg) caused the overexpression of *GFAP* and reduced expression of *BDNF* in brain tissue. Furthermore, AFG1 caused morphological changes in nerve cells and neuroglia and stimulated cell necrosis. There are currently no publications focused on the toxicity of AFG2.		[7,8,9]
**Aflatoxin M1 (AFM1) and M2 (AFM2)**	Generated via the biotransformation of AFB1/AFB2 in the hydroxylation reaction. Derived from animal milk. In mice (16 mg/kg of AFM1), they caused a significant reduction in the weight of the liver and kidneys, increased malondialdehyde (MDA), and reduced glutathione (GSH) levels in the liver and kidneys. Furthermore, AFM1 increased alanine transaminase (ALT), aspartate transaminase (AST), creatinine, and blood urea nitrogen parameters, augmented the formation of micronuclei and chromosomal abnormalities frequencies, and induced DNA fragmentation. There are currently no publications focused on the toxicity of AFM2.		[10]
**Ochratoxin A (OTA) and Ochratoxin B (OTB)**	OTA was shown to be strongly nephrotoxic, hepatotoxic, teratogenic, genotoxic, immunotoxic, and neurotoxic. OTA stimulates apoptosis, necrosis, and the generation of reactive oxygen species (ROS), nitric oxide, and lipid peroxides. Furthermore, OTA inhibits glutathione, cell proliferation processes, and glutamate absorption, which are crucial in the stimulation of neurodegeneration. OTB exhibits significantly lower toxicity compared to OTA. The mechanism of action is very similar; however, the biological effect is diminished.	***Aspergillus*** (*mainly A. ochraceus*, *A. carbonarius*, *A. niger*) ***Penicillium*** (*mainly P. verrucosum*, *P. nordicum*, *P. viridicatum*)	[11,12,13,14,15]
**Patulin**	Strongly hepatotoxic, nephrotoxic, neurotoxic, and immunosuppressive. Highly toxic for the gastrointestinal tract. Stimulates the activity of serum aspartate (AST) and alanine (ALT) transaminase, the generation of ROS, and lipid peroxidation in mice. What is more, patulin induces chromosomal and micronucleus abnormalities. Reduces the GSH level in rat hepatocytes.	***Penicillium*** (*mainly P. expansum*, *P. coprobium*, *P. carneum*, *P. clavigerum*, *P. dipodomyicola*, *P. glandicola*, *P. concentricum*, *P. gladioli*, *P. griseofulvum*, *P. marinum*, *P. paneum*, *P. sclerotigenum*, *P. vulpinum*) ***Aspergillus*** (*mainly A. giganteus*, *A. longivesica*, *A. clavatus*) ***Byssochlamys*** (*B. nivea*) ***Paecilomyces*** (*P. saturatus*)	[16,17,18,19]
**T-2**	Belongs to the Trichothecenes group. Possesses neurotoxic, nephrotoxic, hepatotoxic, and immunotoxic properties. Affects skin, vascular, and reproductive system disorders in humans. Stimulates apoptosis, autophagy, and necrosis in human and animal cells. In mice, the administration of T-2 causes abnormal development of blastocysts, a lower number of blastomeres, and induces chromatin damage. T-2 induces the production of ROS and activation of inflammatory mediators in rat hepatocytes.	***Fusarium*** (*mainly F. sporotrichoides*, *F. poae*, *F. acuminatum*, *F. equiseti*)	[20,21,22]
**Fumonisin**	Immunotoxic, nephrotoxic, hepatotoxic, genotoxic, and neurotoxic. Highly toxic to the heart, lungs, and gastrointestinal tract in humans. Possesses the ability to compete with sphingolipids in ceramide synthase, affecting cell–cell interactions and recognition in humans and animals. Induce soxidative stress by augmenting the production of ROS in animals and humans. Induces DNA damage and epigenetic modifications, i.e., gene methylation. Induces apoptosis and autophagy, and increases the activity of TNF-α and IL-1β.	***Fusarium*** (*mainly F. verticilliodes*, *F. proliferatum*)	[23,24]
**Zearalenone**	Belongs to the Trichothecenes group. Causes hepatotoxicity, genotoxicity, and immunotoxicity. Induces epigenetic alterations, including DNA methylation and histone modifications. Affects crucial pathways associated with metabolism like IGF1, PXR, H2K, and PPARγ in human cells. Inhibits the growth of beneficial microbiota, thus disrupting intestinal balance in mice.	***Fusarium*** (*mainly F. graminearum*, *F. cerealis*, *F. culmorum*, *F. equiseti*, *F. oxysporum*, *F. nivale*)	[25,26,27,28,29]
**Citrinin**	Hepatotoxic, nephrotoxic, immunosuppressive, and carcinogenic in human and animal cell lines. Induces renal mitochondrial dysfunction, and stimulates the production of ROS, causing Balkan nephropathy. May present a synergistic effect in combination with OTA.	***Aspergillus*** (*mainly A. terreus*, *A. niveus*, *oryaze*, *flavus*) ***Penicillium*** (*mainly P. citrinum*, *P. expansum*, *P. radicicola*, *P. verrucosum*, *cameberti*, *notatum*) ***Monascus*** (*mainly M. ruber*, *M. purpureus*	[30,31,32]
